# Functional gait rehabilitation in elderly people following a fall-related hip fracture using a treadmill with visual context: design of a randomized controlled trial

**DOI:** 10.1186/1471-2318-13-34

**Published:** 2013-04-16

**Authors:** Mariëlle W van Ooijen, Melvyn Roerdink, Marga Trekop, Jan Visschedijk, Thomas W Janssen, Peter J Beek

**Affiliations:** 1 MOVE Research Institute Amsterdam, Faculty of Human Movement Sciences, VU University Amsterdam, Van der Boechorststraat 9, Amsterdam, 1081 BT, The Netherlands; 2 Amsterdam Rehabilitation Research Center | Reade, Overtoom 283, Amsterdam, 1054 HW, The Netherlands; 3 PW Janssen, Zorggroep Solis, Hermelijn 2, Deventer, 7423 EJ, The Netherlands

**Keywords:** Gait adaptability, Falling, Older adults, Hip fracture, Treadmill, Exercise

## Abstract

**Background:**

Walking requires gait adjustments in order to walk safely in continually changing environments. Gait adaptability is reduced in older adults, and (near) falls, fall-related hip fractures and fear of falling are common in this population. Most falls occur due to inaccurate foot placement relative to environmental hazards, such as obstacles. The C-Mill is an innovative, instrumented treadmill on which visual context (e.g., obstacles) is projected. The C-Mill is well suited to train foot positioning relative to environmental properties while concurrently utilizing the high-intensity practice benefits associated with conventional treadmill training. The present protocol was designed to examine the efficacy of C-Mill gait adaptability treadmill training for improving walking ability and reducing fall incidence and fear of falling relative to conventional treadmill training and usual care. We hypothesize that C-Mill gait adaptability treadmill training and conventional treadmill training result in better walking ability than usual care due to the enhanced training intensity, with superior effects for C-Mill gait adaptability treadmill training on gait adaptability aspects of walking given the concurrent focus on practicing step adjustments.

**Methods/design:**

The protocol describes a parallel group, single-blind, superiority randomized controlled trial with pre-tests, post-tests, retention-tests and follow-up. Hundred-twenty-six older adults with a recent fall-related hip fracture will be recruited from inpatient rehabilitation care and allocated to six weeks of C-Mill gait adaptability treadmill training (high-intensity, adaptive stepping), conventional treadmill training (high-intensity, repetitive stepping) or usual care physical therapy using block randomization, with allocation concealment by opaque sequentially numbered envelopes. Only data collectors are blind to group allocation. Study parameters related to walking ability will be assessed as primary outcome pre-training, post-training, after 4 weeks retention and 12 months follow-up. Secondary study parameters are measures related to fall incidence, fear of falling and general health.

**Discussion:**

The study will shed light on the relative importance of adaptive versus repetitive stepping and practice intensity for effective intervention programs directed at improving walking ability and reducing fall risk and fear of falling in older adults with a recent fall-related hip fracture, which may help reduce future fall-related health-care costs.

**Trial registration:**

The Netherlands Trial Register (http://NTR3222).

## Background

Falls among older adults are a growing public health problem, particularly in western society with its increasingly aging population. Approximately one third of all community-living people over 65 years fall each year [1-5], and fall incidence rises even further with age [2-6]. Fall incidence is substantially higher in older adults residing in nursing, rehabilitation and hospital care facilities, especially after an injurious fall [6-9]. Falls may lead to fractures, soft tissue injuries and even death [6,10-12]. In The Netherlands, falls among older adults yearly result in 83000 emergency room treatments, 43000 hospital admissions and 2165 deaths [13], which entails an estimated yearly cost of 820M euro [13]. Furthermore, falls are associated with reduced participation, reduced functional ability and fear of falling [6,10,12].

Falls are thus common in older adults and their consequences and costs are well documented. Falls in older adults mostly occur during walking [9,11] and are caused by a combination of intrinsic and extrinsic factors. Intrinsic factors associated with falling are generally age-related and include reduced executive function, increased gait-related attentional demands, decreased muscle strength, impaired vision, and gait and balance impairments [1,14-20]. Extrinsic risk factors for falling include poor lighting conditions, inappropriate footwear and environmental hazards such as obstacles, cluttered terrain, the presence of pets and slippery surfaces [1,6,11]. These environmental hazards contribute to approximately half of all falls [1,6,11,21,22]. Therefore, gait adaptability (i.e., the ability to adjust gait to environmental hazards; [23]) evidently seems an important factor to include in intervention programs aimed at reducing falls in older adults.

Strikingly, however, intervention programs rarely incorporate gait adaptability as an explicit target to reduce falls and fall risk in older adults (see Weerdesteyn et al. [24] for a notable exception), despite its self-evident importance. Instead, fall-prevention programs have focused on other factors, including vitamin D supplementation, medication optimisation, education, multi-factorial interventions, environmental home safety interventions and exercise programs [5]. Although multi-factorial interventions appear effective in reducing fall rate, beneficial effects for reducing fall risk as well as fall rate in community dwelling older adults are only established for exercise programs and environmental home safety interventions [5]. The removal of environmental hazards in the house is thus an effective form of fall prevention, which further adds to the importance of environmental hazards as risk factor for falls, and highlights the need for exercise programs that integrate the environmental hazards encountered in every-day life (i.e., gait adaptability).

**Figure 1:**
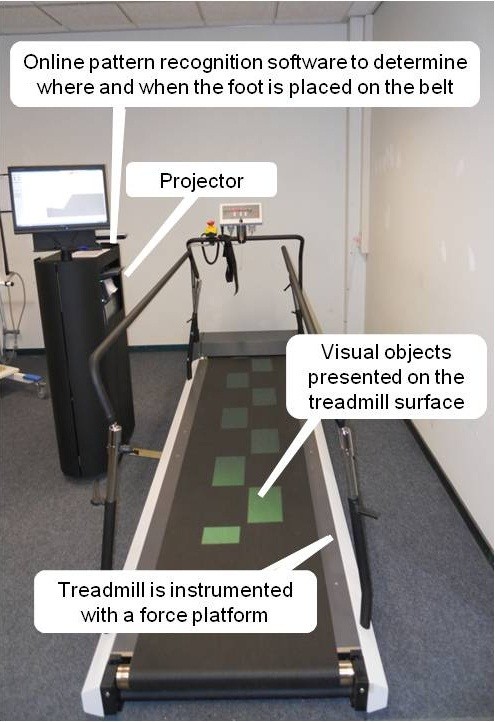
**C-Mill.** The C-Mill is a 3-m long instrumented treadmill with visual objects such as stepping targets and obstacles projected on the belt to facilitate practice of foot placement relative to environmental context. A force platform is embedded in the treadmill for real-time determination of gait and foot-placement positions, allowing for various gait adaptability interventions accustomed to an individual’s gait. The visual context is projected on the belt’s surface, approaching the patient with the speed of the treadmill belt. The handrails permit weight-bearing and allow early training in a safe environment.

The fact that gait adaptability is generally not targeted in interventions aimed at reducing falls and fall risk in older adults implies that there is ample room for improvement of fall-prevention programs. The recent introduction of the C-Mill (Figure 1; ForceLink, Culemborg, The Netherlands) holds significant promise in that regard. The C-Mill is an innovative, instrumented rehabilitation treadmill with task-relevant visual context (e.g., obstacles, stepping targets) projected on the belt’s surface. The C-Mill was explicitly developed as a therapeutic tool to practice gait adaptability, elicited by aligning foot placement relative to the projected visual context [25]. In other words, the presented obstacles and stepping targets on the belt’s surface mandate gait adjustments to accurately position the feet relative to that visual context, akin to the gait adjustments required to walk safely in continually changing everyday environments. Because the C-Mill is instrumented with a force platform, it is known when and where the feet are placed on the belt’s surface [26], allowing for direct performance feedback with regard to gait parameters (e.g., step width, step length) as well as gait-context interactions (e.g., foot placement relative to the presented visual context; see Methods for more details). Whereas C-Mill training is focused explicitly on practicing gait adaptability relative to environmental hazards, it may as well implicitly target various intrinsic and extrinsic factors associated with falling, such as executive function [14,17,27], gait-related attentional demands [17,27-29], postural control during walking and step adjustments [30,31], and muscle strength [31-33].

This study protocol is designed to examine the efficacy of C-Mill gait adaptability training for improving walking ability and reducing fall incidence and fear of falling in older adults residing in inpatient rehabilitation care to recover from a fall-related hip fracture, a group particularly prone to falling [6-9]. The efficacy of C-Mill gait adaptability training will not only be contrasted against usual care, comprising of exercises in strength, balance, overground walking, transfers and activities of daily living, but also against intensity matched conventional treadmill training (i.e., without visual context presented on the belt), promoting repetitive stepping. Literature on conventional treadmill training in older adults recovering from hip injury is quite scarce, but generally positive with regard to its efficacy [32,33]. Conventional treadmill training outperforms overground gait training in that regard, most likely because of the enhanced intensity of training (i.e., defined in number of steps taken per training session). Older adults with a total hip arthroplasty, for example, performed 1000-1500 steps during a conventional treadmill training session compared to 100-150 steps during an overground gait training session of similar duration [32]. We therefore hypothesize that both conventional treadmill training and C-Mill gait adaptability treadmill training result in better outcomes related to walking ability than usual care due to the enhanced training intensity, with superior effects for C-Mill gait adaptability treadmill training on gait adaptability aspects of walking given the concurrent focus on practicing gait adaptability. The present study will thus shed light on the relative importance of high versus low practice intensity and adaptive versus repetitive stepping as key ingredients for effective intervention programs aimed at improving walking ability and reducing fall risk and fear of falling in older adults recovering from a fall-related hip fracture.

## Methods/design

### Recruitment

All patients with a hip fracture admitted to residential and rehabilitation centre Zorggroep Solis, Deventer, The Netherlands will be assessed for eligibility within 3 days from admission by a physical therapist during regular intake. We aim to recruit 126 geriatric patients. Inclusion criteria are admission with a hip fracture related to falling, age ≥ 65 years, Functional Ambulation Category score 2 or higher (FAC, [34,35]), expected duration of admission ≥ 6 weeks and an ability to understand and execute simple instructions. Exclusion criteria are not being allowed to bear weight on the affected leg, moderate or severe cognitive impairments as indicated with a score below 18 at the Mini-Mental State Examination (MMSE, [36]), severe non-corrected visual impairments, contraindication to physical activity and an activity tolerance below 40 minutes with rest intervals. Patients eligible for participation will be informed of the current study by the physical therapist, both verbally and in writing. Patients who are willing to participate will be asked to give informed consent.

### Ethical approval

This study has been approved by the Medical Ethical Reviewing Committee of VU University Medical Centre, Amsterdam, The Netherlands (cf., protocol number 2011/327 and Central Committee on Research Involving Human Subjects, CCMO, protocol number NL37842.029.11).

### Study design

**Figure 2:**
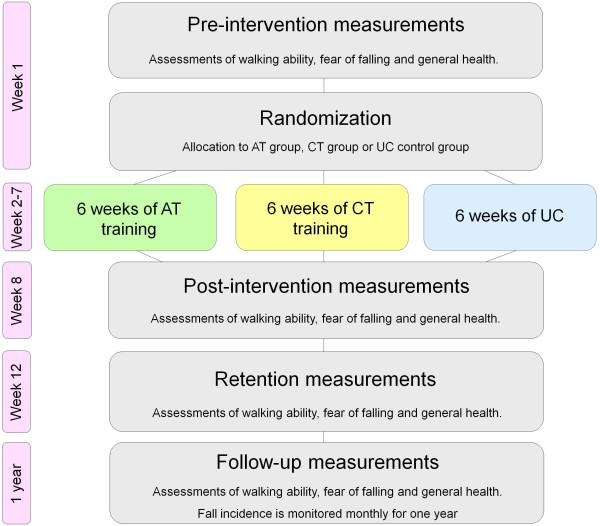
**Flow chart of the study procedures.** Abbreviations: AT = adaptability treadmill, CT = conventional treadmill, UC = usual care.

This protocol presents a parallel group, single-blind, superiority randomized controlled trial with pre-tests, post-tests, retention-tests and follow-up (Figure 2) to evaluate the relative efficacy of three intervention programs. Prior to randomization, pre-intervention assessments (T0) will be performed by one of two assessors within one week from informed consent. Subsequently, the assessor will randomly allocate (ratio 1:1:1) participants to one of three groups: 1) usual care (UC) control group, 2) conventional treadmill (CT) intervention group, 3) adaptability treadmill (AT) intervention group. We will use computer-generated block randomization with a block size of 21 participants to ensure equal group size after each block, with allocation concealment by opaque sequentially numbered envelopes that will be allocated serially to participants. An independent assessor blinded to group allocation will conduct post-intervention assessments (T1) within one week after completion of the intervention program. The same assessor will repeat these assessments 1 month later (retention assessments, T2). Follow-up assessments (T3) will be performed 12 months after completion of the intervention program. Furthermore, the incidence of trips, slips and falls will be monitored monthly between T1 and T3.

### Intervention program

The intervention programs will be dose-matched in terms of duration (i.e., 40 minutes per session) and frequency (i.e., five sessions per week) of therapy over a 6-week inpatient intervention period. All intervention programs will be provided by physical therapists of Solis Zorggroep, Deventer, The Netherlands. Therapy sessions will typically be conducted by a single physical therapist with two participants per session; the two participants alternately exercise and rest, resulting in 20 minutes of practice per session. Physical therapists and participants cannot be blinded to group allocation.

In the *usual care* (UC) control group, participants will receive conventional physical therapy in all 30 sessions, consisting of exercises of upper-leg strength, balance, transfers, overground walking, and activities of daily living, following locally implemented guidelines of Solis Zorggroep, Deventer, The Netherlands and Deventer Hospital, Deventer, The Netherlands regarding the treatment of hip fractures.

In the *conventional treadmill* (CT) intervention group, participants will receive 15 conventional physical therapy sessions (as in UC) and 15 conventional treadmill training sessions (week 1-3: 2 per week, week 4-6: 3 per week) with an emphasis on repetitive stepping. Participants will walk at comfortable treadmill walking speed, without body-weight support other than handrail support. Comfortable treadmill walking speed will be determined at the beginning of each session in order to promote the quality and safety of walking as appraised by the physical therapist. The initial focus of conventional treadmill training will be on the quality and safety aspects of walking: therefore, therapists may give instructions regarding the walking pattern. After a safe walking pattern has been established, the focus will gradually shift towards walking faster and longer. Participants in the CT group will walk on the C-Mill treadmill (C-Mill, ForceLink, Culemborg, The Netherlands), but without projection of visual context on the belt’s surface.

In the *adaptability treadmill* (AT) intervention group, participants will receive 15 conventional physical therapy sessions (as in UC) and 15 C-Mill gait adaptability treadmill training sessions (week 1-3: 2 per week, week 4-6: 3 per week) with an emphasis on practicing gait adaptability. As in CT, participants will walk at comfortable treadmill walking speed, without body-weight support other than handrail support. The two AT training sessions in the first week will consist of conventional treadmill training with an emphasis on the safety and quality aspects of walking (i.e., without visual context, similar to CT), so that participants will become acquainted to treadmill walking.

**Figure 3:**
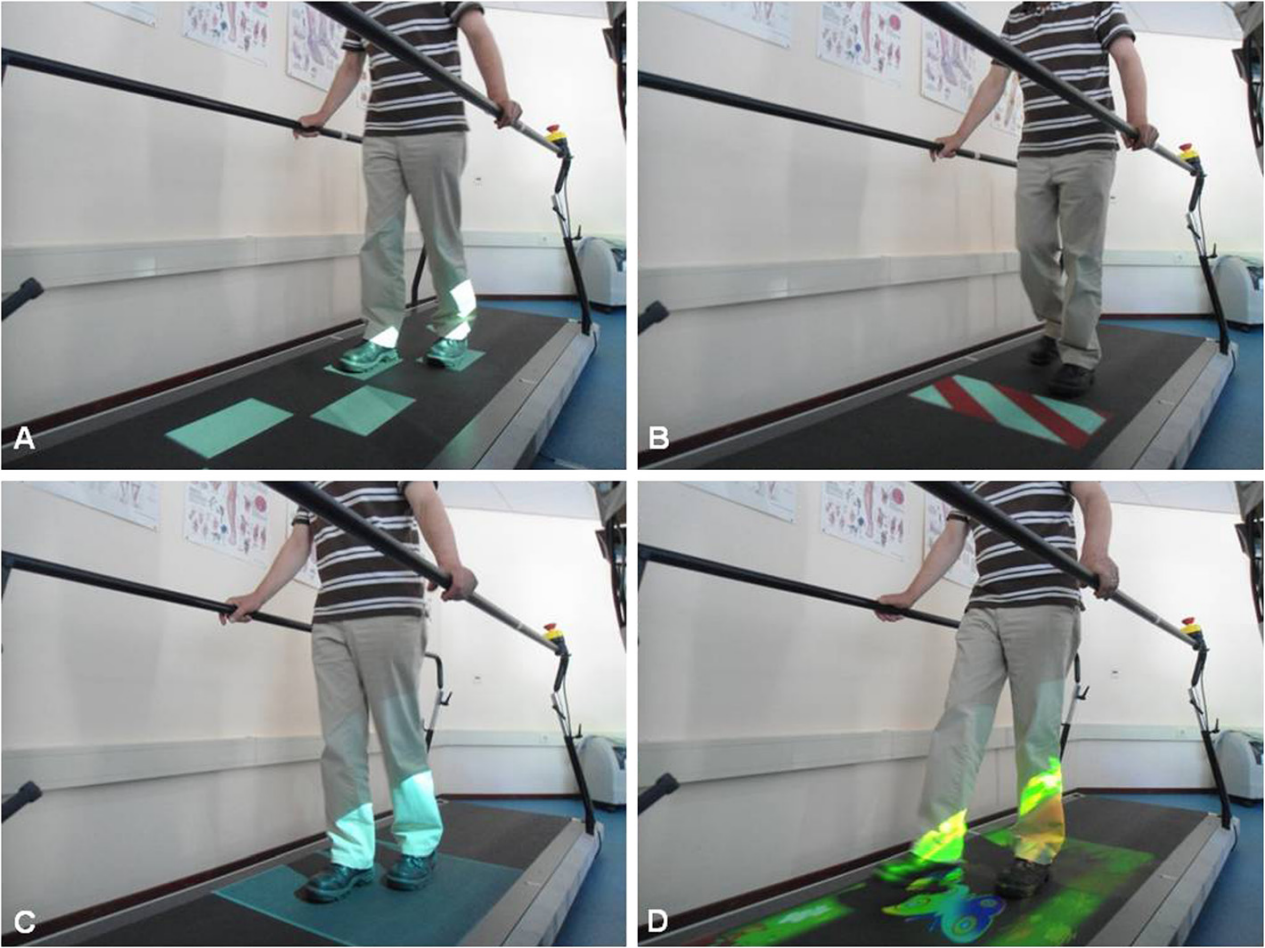
**Examples of C-Mill gait adaptability exercises.** Participants practice visually guided stepping to a sequence of regular or irregular stepping targets (**A**), obstacle avoidance (**B**), speeding up and slowing down by maintaining position in a moving walking zone (**C**), and all of the above in a functional and interactive gait adaptability game (**D**).

From week 2 onwards, AT training sessions progressively utilize the C-Mill’s visual context to elicit step adjustments (Figure 3 and Additional file 1). The gait adaptability exercises will be increased in difficulty from week to week to ensure that they remain sufficiently challenging. For reasons of safety and feasibility this will be done in a controlled fashion, tailored to the participant’s progress and ability. Specifically, in the second week of AT training, visually guided stepping to a sequence of stepping targets (Figure 3A) will be introduced to practice foot positioning relative to the environmental context projected on the belt. The spatial configuration of the sequence of stepping targets can be manipulated, requiring gait adjustments in terms of step length, step width and step-length symmetry. Furthermore, step adjustments can also be elicited on a step-to-step basis by introducing a degree of irregularity in the sequence of stepping targets. Consequently, each step needs to be planned and executed from scratch to ensure accurate foot placement relative to the stepping target. Visually guided stepping can be made more challenging by changing the degree of irregularity in the sequence of stepping targets and by scaling their size to the participants’ feet. In the third week, obstacle avoidance exercises will be included (Figure 3B) while continuing with the visually guided stepping exercises. Visual obstacles are projected onto the treadmill belt, and the difficulty level for obstacle avoidance will be controlled by the physical therapist by changing the size of the obstacle as well as the available response time (i.e., obstacles can appear a few steps ahead [easy] or during the swing phase of an ongoing step [difficult]). Obstacle avoidance is also evoked in the visually guided stepping exercises by introducing sudden target to obstacle shifts in the sequence of stepping targets, requiring an adjustment of an already planned step. From the fourth week onwards, participants will also practice speeding up and slowing down. To this end, a projected walking area of approximately 1 m^2^ will oscillate in anterior-posterior direction over the treadmill surface (Figure 3C). Participants will need to accelerate or decelerate relative to the fixed belt speed in order to follow the walking area; therapists can progressively or randomly vary the acceleration of the walking area to increase the difficulty of this speeding-up/slowing-down exercise. From the fourth week onwards, also so-called gait adaptability games will be introduced in the AT training sessions (Figure 3D). These gait adaptability games consist of interactive forest or beach trails scattered with stepping targets (e.g., beach balls) and obstacles (e.g., seals, shells, crabs). Participants can score or lose points depending on the successfulness of foot positioning relative to this environmental context. The interaction with the environment will be intensified further by means of direct feedback of stepping performance (e.g., the beach ball will pop forward if participants successfully step on it while a seal will scream loudly if participants accidentally step on it).

Note that we predict that the intensity of training, in terms of number of performed steps, will be markedly higher for both treadmill training intervention groups (i.e., AT and CT) than for the UC control group. We will test this prediction by comparing the number of steps taken per session among groups. To this end, the number of steps taken during AT and CT training sessions will be registered using the treadmill’s step counter while an observer will count the number of steps taken during UC sessions in a random sample of participants. We expect a main effect of Group in a one-way ANOVA, with post-hoc independent *t*-tests revealing significantly fewer steps for the UC control group than for both AT and CT training groups, in the absence of a significant difference between the latter groups.

To assist the therapist in progressively scaling the AT and CT training sessions, the participant’s perceived rate of fear and difficulty during AT and CT sessions will be assessed at a scale from 0 (no fear/not difficult) to 10 (much fear/very difficult), as well as their perceived rate of exertion using the 15 grade Borg scale (range 6-20, [37]).

Finally, adherence will be reported and the content of all individual training sessions will be registered by the supervising physical therapist. Adverse events during training sessions will be reported as well, and after completing the last UC, CT or AT therapy session of the intervention period, participants will fill out a purpose-designed questionnaire to register perceived discomforts during and after training sessions. The participant’s experience with the therapy program will be evaluated with this questionnaire as well to be able to compare the feasibility of the interventions from a participant’s perspective.

### Outcome measures

Measures related to walking ability are the primary outcome in this study, whereas measures related to fear of falling, fall incidence and general health are secondary outcomes. Table 1 provides an overview of the assessments performed at T0, T1, T2 and T3.

**Table 1:** Study assessment schedule

		**Intake**	**T0**	**T1**	**T2**	**T3**
**Primary outcome measures related to walking ability**						
Performance Oriented Mobility Assessment (POMA)			x	x	x	x
10 Meter Walking Test with obstacles (10MWT_obstacle_)				x	x	
10 Meter Walking Test with cognitive task (10MWT_cognitive_)				x	x	
Trail Making Test (TMT)			x	x	x	x
Functional Ambulation Category (FAC)		x	x	x	x	x
Elderly Mobility Scale (EMS)			x	x	x	x
10 Meter Walking Test (10MWT)				x	x	
Timed Up-and-Go test (TUG)				x	x	
Nottingham Extended Activities of Daily Living (NEADL)			x		x	x
**Secondary outcome measure, related to fear of falling**						
Falls Efficacy Scale International (FES-I)			x	x	x	x
**Secondary outcome measure, related to fall incidence**						
Monthly fall diary						x
**Secondary outcome measures, related to general health**						
Visual Analogue Scale of perceived general health (VAS)			x	x	x	x
Mini-Mental State Examination (MMSE)		x				
Hip Disability and Osteoarthritis Score (HOOS)					x	x

#### T0 (pre-intervention assessments)

Outcome measures related to walking ability obtained during T0 will comprise scores of mobility (POMA [38-40], Elderly Mobility Scale EMS; [41,42]), executive function (Trail Making Test TMT; [43-45], requiring visual scanning, task shifting, planning and mental flexibility), general functional walking ability (Functional Ambulation Category FAC; [34,35]), and pre-admission activity of daily living status (Nottingham Extended Activities of Daily Living scale NEADL; [46-48]). Outcome measures related to fear of falling and general health will include perceived fear of falling (Falls Efficacy Scale International FES-I; [49,50]) and perceived general health status (visual analogue scale VAS; [51]). Demographics, medical information regarding hip fracture, co-morbidities, medication use and pre-admission daily functioning, including the use of assistive devices, will be obtained from medical files.

#### T1 (post-intervention assessments)

Similar to T0, POMA, EMS, TMT, FAC, FES-I and VAS scores of perceived general health will be obtained at T1 as well. Current living situation and the use of assistive devices will also be scored. To evaluate walking ability, we will further perform the Timed Up-and-Go test (TUG, [52]) and the 10 Meter Walk Test (10MWT, [48]). In addition to the standard 10MWT, we will also perform the 10MWT with 3 obstacles in the walkway to evaluate obstacle avoidance during walking (10MWT_obstacle_; one 10 × 20 × 5 cm and two 5 × 20 × 10 cm obstacles [height × width × length]) and while subtracting 3’s from a random number between 191 and 199 to evaluate the dual-task costs of walking (10MWT_cognitive_; the serial-3 subtraction task will first be practiced for 60 seconds). Accordingly, the serial-3 subtraction task will also be performed as a single task. The modified 10MWTs are deemed informative given the older adults’ impaired ability to avoid obstacles and their elevated attentional demands of walking – both factors related to fall risk [17,24,53-55]. The TUG and 10MWTs will be performed twice, taking the average time in seconds that is necessary to complete each test as the outcome measure. The number of serial-3 subtractions as well as the number of mistakes made will be used as additional outcome measures for evaluating the dual-task costs of walking.

#### T2 (retention assessments)

All tests performed at T1 will be repeated 4 weeks later at T2. In addition, the NEADL will be conducted to assess the activities of daily living performed between T1 and T2. The Hip Disability and Osteoarthritis Score (HOOS, [56,57]) will be administered to monitor symptoms and functional limitations related to the hip. In addition, participants will be asked about the use of assistive devices, tolerated walking distance, complications and physical therapy in the past four weeks. If applicable, the content of physical therapy will be recorded.

#### T3 (follow-up assessments)

All tests performed at T0 will be repeated after 12 months follow-up at T3 and participants will again be questioned about the use of assistive devices, tolerated walking distance, complications and physical therapy in the past 12 months. The HOOS will also be administered at T3. Furthermore, the occurrence of trips, slips and falls will be monitored monthly between T1 and T3 using a daily calendar diary for postal use [58]. When a fall occurs, the participant has to answer additional questions on the daily calendar diary regarding the circumstances of the fall and the injuries caused by the fall. If a calendar is not returned or information is incomplete, the participant will be reminded to return the calendar or the missing information will be obtained during a telephone conversation.

### Sample size

We used the POMA [38] to perform a sample size calculation, because the POMA is a generic and widely used test to evaluate walking ability and has been associated with risk of falling [39,40]. Earlier clinical trials in which the POMA was used as an outcome measure reported mean differences of 4.0 and 3.5 POMA points between the intervention and control group [59,60]. The average standard deviations at follow-up measurements in these studies were respectively 3.8 and 4.8 POMA points. Assuming the lowest difference in mean value between groups (i.e., 3.5 POMA points), the highest standard deviation (i.e., 4.8 POMA points) and a correlation coefficient of 0.7, a sample size of 32 participants in each group is required to achieve 80% power with a two-tailed conservative alpha of 0.017 that is corrected for multiple comparisons. This sample size is based on the formula given by Twisk [61] to compare longitudinal outcome measures between groups.

We will include 126 participants who complete at least four weeks of intervention and pre- and post-intervention assessments (i.e., T0 and T1). Hence, participants who do not adhere to the protocol will be replaced to maintain a valid per-protocol assessment of sufficient power. In addition, this sample size allows for a maximum drop-out rate of 24% from T1 to T3.

### Data analyses

Descriptive statistics will be used to present group characteristics (e.g., sex, age, MMSE, medication use, comorbidities, side of fracture and the use of an assistive device), therapy adherence and adverse events. Baseline characteristics will be compared between groups with one-way ANOVAs. Given the explanatory objective of this study regarding the efficacy of C-Mill gait adaptability training, data will be analyzed per protocol, which implies that we will only include data of participants who completed at least 4 weeks of training and pre-intervention and post-intervention assessments (i.e., both T0 and T1). For longitudinal outcome measures (FAC, POMA, EMS, VAS, FES-I, TMT, NEADL, 10MWT, 10MWT_cognitive_, 10MWT_obstacle_, TUG, HOOS), multilevel regression analyses will be applied, which is appropriate for analyzing longitudinal data in which observations within one participant over time are correlated [61]. In addition, multilevel regression analyses account for missing values and allow baseline covariates to control for potential differences in baseline characteristics between groups [61].

## Discussion

Falls are a common mishap among older adults and impose a major burden on older adults and the community at large; it is therefore important to gather evidence on the effectiveness of intervention programs and their design features (e.g., adaptive versus repetitive stepping, high versus low practice intensity). The current study was conceived to examine the efficacy of gait adaptability exercises in intervention programs aimed at improving walking ability and reducing fall incidence. Recently, encouraging first steps in this direction have been taken (e.g., [24,27,62,63]). Weerdesteyn et al. [24], for example, integrated overground gait adaptability exercises in a 5-week Nijmegen Falls Prevention Program for older adults, and found improved obstacle avoidance success rates, improved balance confidence and reduced fall incidence during one year follow-up. Given that the Nijmegen Falls Prevention Program consisted of overground walking, and hence practice intensity was presumably fairly low (i.e., number of steps taken per session), the observed beneficial effects are encouraging as they may be enhanced even further by increasing the practice intensity of gait adaptability exercises, such as with our C-Mill based gait adaptability training protocol.

In another encouraging study, Mirelman et al. [27] exploited the potential of virtual reality (VR) to practice step adjustments while walking on a treadmill. Participants’ feet positions were fed back in a virtual environment (i.e., a path with obstacles and targets) presented on a monitor in front of them. After 6 weeks of VR treadmill training, improved obstacle avoidance behavior, higher walking speed while performing a dual task and improved executive function (as assessed with the TMT) were observed for people with Parkinson’s disease. Also for people in the chronic phase after stroke, beneficial effects of VR treadmill training, using a head-mounted display [62] or a large screen in front of the treadmill [63], have been reported. All of these studies indicated that gait adaptability training with VR may help improve walking ability and reduce fall incidence in populations prone to falling. Note that C-Mill gait adaptability training is distinct from VR gait adaptability training in that C-Mill training elicits gait adjustments relative to visual context presented in the real environment, whereas with virtual reality training gait adjustments in the real world are detached from the context presented in the virtual environment. Given that gait adjustment is tied to task-relevant visual context in the VR environment only, VR gait adaptability training fails to utilize the direct visuomotor coupling of walking (e.g., [64-66]), where point of gaze is coupled to future foot placement locations, particularly in the presence of surface irregularities. Moreover, visuomotor control of targeted stepping deteriorates with aging, resulting in less accurate foot placement relative to stepping targets, especially in elderly with a high fall risk [67,68], but proved to ameliorate with training [69]. These examples illustrate the intimate relation between point of gaze and stepping accuracy relative to environmental context. The direct coupling between gaze and gait is fully exploited in C-Mill gait adaptability treadmill training in which step adjustments are elicited relative to visual context in the real environment (viz. augmented reality) rather than visual context in the virtual environment (viz. virtual reality). C-Mill gait adaptability training may thereby tentatively be even more effective than the abovementioned VR applications to practice step adjustments [27,62,63], which lack such a direct coupling.

The expected superior outcome of C-Mill gait adaptability treadmill training relative to conventional treadmill training is further supported by current insights into motor learning in the context of neurorehabilitation [70], which suggest that a considerable variability during practice enhances long-term training effects and transfer to new tasks and contexts compared to constant and repetitive practice (e.g., conventional treadmill training; [71-73]). Several theories of motor learning stress the importance of variability in training, including Schmidt’s schema theory [74], and theories of contextual interference [75] and differential learning [76]. These theoretical approaches have in common that one should exploit rather than eliminate variation in task performance to yield optimal learning effects in terms of retention and transfer. This insight is highly relevant for rehabilitation, which aims at long-lasting and general improvements. Exercises during C-Mill gait adaptability treadmill training, such as obstacle avoidance, visually guided stepping, speeding-up and slowing-down, and the interactive gait adaptability games, promote variable practice, which is further reinforced by varying obstacle size, moment of obstacle presentation, and the amount of variability in visually guided stepping (see Methods, Intervention program; see Additional file 1). This variable practice environment affords deep motor learning in that each step needs to be planned and executed from scratch in order to position it successfully relative to the visual context projected on the belt’s surface (e.g., [75]).

Gait adjustments to environmental context strongly rely on executive function [16,17], which comprises multiple cognitive processes including visual scanning, problem solving, planning, and task shifting (cf. the deeper form of motor learning referred to above). Interestingly, previous studies found indications for improved executive function after VR treadmill training with an emphasis on step adjustments [27], as measured with the Trail Making Test [43-45]. Such training induced changes in executive function are important because reduced executive function has been associated with gait impairments, reduced obstacle avoidance ability and falling [14,15,17]. Moreover, executive function is known to decline with age [17]. Considering that visual scanning, problem solving, planning, and task-shifting are integral elements in C-Mill gait adaptability treadmill training (i.e., to secure adequate foot placement relative to the projected environmental context), similar training induced improvements in executive function –which will be quantified by the Trail Making Test– may be observed in the present study for AT compared to CT and UC groups.

### Limitations

The inclusion and exclusion criteria for this study are deliberately quite broadly defined, such that the included patients are likely to be representative of the older population seen in daily clinical practice. This implies that the included patients likely exhibit various co-morbidities, resulting in heterogeneous study groups akin to the general population of older adults. On the one hand this may hamper between-group comparisons, but on the other hand findings may generalize well to mixed populations of older adults, which likely facilitates future implementation of the study’s results.

The use of vitamin D will not be intervened in the current study, implying that participants will not be supplemented with vitamin D as a standard. This resembles daily clinical practice, and may therefore also facilitate future implementation of the study’s results. However, since vitamin D supplementation has been associated with reduced fall rate and risk [5,7], supplementation will be reported in order to be able to control for vitamin D supplementation afterwards. Likewise, smoking history will be registered, since smoking is likely to adversely affect bone healing, bone mineral density, wound healing and the incidence of hip fractures [77-80].

Some outcome measures of walking ability (i.e., TUG, 10MWT, 10MWT_cognitive_ and 10MWT_obstacle_) will not be assessed at T0, mainly because pain and exertion will limit the number and content of conductible tests at T0. Comparing outcome measures not assessed at T0 between groups entails the risky assumption that groups do not differ at T0. To test this assumption, POMA, FAC and EMS, which are good indicators of walking ability, will be examined at T0. Results based on outcome measures not assessed at T0 should be interpreted with care. Other points of consideration include the different assessors at T0, T1, T2 and T3, and the different setting of T3, which is administered during a home visit. Although the performed tests are well standardized and have good validity and reliability [35,39,42-44,47,50,51,57], differences in test setting should be kept in mind when interpreting the results. The limitations in the current study mostly concern logistic choices based on clinical constraints, which are handled as well as possible to minimize bias.

## Conclusions

The study will provide insight into the effect of C-Mill gait adaptability treadmill training and conventional treadmill training for improving walking ability and reducing fall risk and fear of falling in older adults recovering from a fall-related hip fracture. In this regard, the results of this study are likely to contribute to the effectiveness of intervention programs. Moreover, the study will shed light on the relative importance of adaptive versus repetitive stepping and high versus low intensity of practice as ingredients for fall prevention programs, and may help reduce future fall-related health-care costs. The results of this study are expected in 2015.

## Competing interests

MR and PJB are inventors of rehabilitation treadmills that include visual context for foot placement (cf. [25]). VU University Amsterdam granted this idea exclusively to ForceLink (Culemborg, The Netherlands), an industrial partner of VU University Amsterdam. ForceLink is manufacturer of the C-Mill and assignee of a patent for rehabilitation treadmills with visual context for foot placement, with MR and PJB listed as inventors. VU University Amsterdam received patent revenues, and transferred part of these revenues to spend them freely for their research endeavors. VU University Amsterdam used these revenues to finance a research project on the effectiveness of C-Mill training. The current study is part of that research project. MR and PJB did not receive reimbursements, fees, funding or salary from ForceLink, nor do they benefit personally from patent revenues.

## Authors’ contributions

MWO is the executive investigator, prepared the first draft of the paper, participated in designing the study and treatment protocols and coordinates the study in Zorggroep Solis, Deventer. MR revised the manuscript critically, conceived the study, participated in designing the study and treatment protocols, and coordinates the study and its financial support. MT participated in designing the study and treatment protocols, and coordinates the study and recruitment in Zorggroep Solis, Deventer. JV participated in designing the study and treatment protocols and coordinates the collection of medical information. TWJ revised the manuscript critically, participated in its design and conceived the study and its coordination. PJB revised the manuscript critically, conceived the study, participated in its design and coordinates the study and its financial support. All authors read and approved the manuscript.

## Pre-publication history

The pre-publication history for this paper can be accessed here:

http://www.biomedcentral.com/1471-2318/13/34/prepub

## Supplementary Material

Additional file 1 **Video of C-Mill gait adaptability exercises.** C-Mill gait adaptability exercises include visually guided stepping to a regular or irregular sequence of stepping targets, obstacle avoidance, speeding up and slowing down, and all of the above in a functional and interactive gait adaptability game. Click here for file
